# Lifespan extension by increased expression of the *Drosophila* homologue of the IGFBP7 tumour suppressor

**DOI:** 10.1111/j.1474-9726.2010.00653.x

**Published:** 2011-02

**Authors:** Nazif Alic, Matthew P Hoddinott, Giovanna Vinti, Linda Partridge

**Affiliations:** Institute of Healthy Ageing, and GEE, University College LondonDarwin Building, Gower Street, London WC1E 6BT, UK

**Keywords:** aging, *Drosophila*, IMP-L2, insulin/insulin-like growth factor signalling, insulin-like growth factor-binding protein

## Abstract

Mammals possess multiple insulin-like growth factor (IGF) binding proteins (IGFBPs), and related proteins, that modulate the activity of insulin/IGF signalling (IIS), a conserved neuroendocrine signalling pathway that affects animal lifespan. Here, we examine if increased levels of an IGFBP-like protein can extend lifespan, using *Drosophila* as the model organism. We demonstrate that Imaginal morphogenesis protein-Late 2 (IMP-L2), a secreted protein and the fly homologue of the human IGFBP7 tumour suppressor, is capable of binding at least two of the seven *Drosophila* insulin-like peptides (DILPs), namely native DILP2 and DILP5 as present in the adult fly. Increased expression of *Imp-L2* results in phenotypic changes in the adult consistent with down-regulation of IIS, including accumulation of eIF-4E binding protein mRNA, increase in storage lipids, reduced fecundity and enhanced oxidative stress resistance. Increased *Imp-L2* results in up-regulation of *dilp2*, *dilp3* and *dilp5* mRNA, revealing a feedback circuit that is mediated via the fly gut and/or fat body. Importantly, over-expression of *Imp-L2*, ubiquitous or restricted to DILP-producing cells or gut and fat body, extends lifespan. This enhanced longevity can also be observed upon adult-onset induction of *Imp-L2*, indicating it is not attributable to developmental changes. Our findings point to the possibility that an IGFBP or a related protein, such as IGFBP7, plays a role in mammalian aging.

## Introduction

The insulin/insulin-like growth factor (IGF) signalling (IIS) pathway is an evolutionarily conserved neuroendocrine signalling pathway that controls a variety of processes and traits in animals, including growth and development, energy metabolism, reproduction and stress resistance. Genetic manipulations of pathway components that result in dampened IIS extend lifespan in worms, flies and mice, and ameliorate age-dependent functional decline (Tatar *et al.*, 2003; Piper *et al.*, 2008). Genetic variation in several components of this pathway is strongly associated with human longevity (Kuningas *et al.*, 2007; Willcox *et al.*, 2008; Flachsbart *et al.*, 2009; Pawlikowska *et al.*, 2009), confirming the relevance of IIS to human aging.

Central to the pathway are insulin-like ligands, which include insulin, IGF-I and IGF-II in mammals (White, 2006); 38 insulin-like peptides in worms (Pierce *et al.*, 2001) and the seven *Drosophila* insulin-like peptide (DILPs) in flies (Brogiolo *et al.*, 2001). The ligands mediate cell-to-cell signalling by activating an insulin receptor-like receptor, leading to the activation of PI3-kinase – Akt, TOR and ERK intracellular signalling pathways (Tatar *et al.*, 2003; White, 2006; Piper *et al.*, 2008). Importantly, not all manipulations of the pathway result in lifespan extension (Clancy *et al.*, 2001; Tatar *et al.*, 2001; Hwangbo *et al.*, 2004; Selman *et al.*, 2008; Ikeya *et al.*, 2009), suggesting that the pathway needs to be manipulated to a specific level of signal reduction and in specific tissues to achieve enhanced longevity.

In mammals, a layer of complexity is added to IIS by the presence of IGF binding proteins (IGFBPs). Six classic IGFBPs bind IGF-I and IGF-II with high affinity and act as modulators of IGF activity. They can both enhance and dampen IIS by prolonging the half-life of IGFs, altering their local and systemic availability and preventing them from binding to the receptor (Hwa *et al.*, 1999). Furthermore, mammals possess IGFBP-related proteins, such as IGFBP7, that bind IGFs with lower affinity (Hwa *et al.*, 1999). Notably, IGFBP7 has received attention as a potent secreted tumour suppressor acting in an autocrine/paracrine manner to block melanoma genesis (Wajapeyee *et al.*, 2008).

Insects also possess IGFBP-like proteins, the first of which was discovered serendipitously, allowing the subsequent identification of the *Drosophila* Imaginal morphogenesis protein-Late 2 (*Imp-L2*) (Sloth Andersen *et al.*, 2000; Alic & Partridge, 2008). IMP-L2 resembles IGFBP7 in sequence and it appears to be equivalent to an IGFBP in function, acting as a negative regulator of IIS during development, regulating growth cell-non-autonomously and antagonising *dilp2* in genetic assays (Honegger *et al.*, 2008). Interestingly, when the germline is ablated late in fly development, lifespan is increased with a concomitant increase in the levels of *Imp-L2* mRNA (Flatt *et al.*, 2008), indicating that one of the roles of *Imp-L2* may be to mediate a lifespan-extending signal emanating from the gonad to IIS in the soma. However, the role of this gene in IIS in the adult fly, including its capacity to enhance longevity, has not been examined.

While the complex and important effects of IGFBPs on IIS in mammals and, in turn, the role of IIS in animal lifespan are both well established, no study has examined if IGFBPs or related proteins can alter animal physiology in such a way as to enhance longevity. To determine if increased function of an IGFBP can extend lifespan, we used *Drosophila* as a model; such an approach has been fruitful in the past, when the finding that a mutation in an insulin-receptor substrate extends lifespan in the fly (Clancy *et al.*, 2001) was subsequently confirmed in mammals (Taguchi *et al.*, 2007; Selman *et al.*, 2008). Here, we show that the fly IGFBP homologue, IMP-L2, binds native DILP2 and DILP5, is involved in a feedback circuit with *dilp2*, *dilp3* and *dilp5*, affects IIS-regulated traits in the adult and extends lifespan, pointing to the possibility that an IGFBP or a related protein could modulate mammalian aging.

## Results

### Eighty percent increase in *Imp-L2* activates transcription of *4E-BP*

**Fig. 1:**
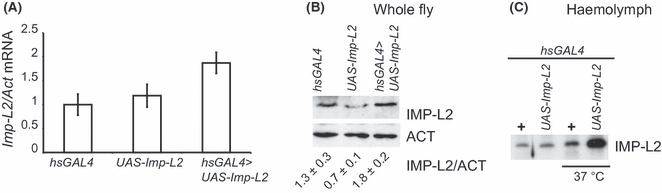
Over-expression of *Imp-L2* with *heatshockGAL4* driver. The flies were reared at 25°C and harvested on day 7. (A) The transcript levels of *Imp-L2* were determined by qPCR, normalised to *Act* mRNA and the ratio in *hsGAL4* set to one. Means and standard errors are shown with *n* = 6 for *hsGAL4 > UAS-Imp-L2* and *n* = 7 for the two controls. The measurements for *hsGAL4 > UAS-Imp-L2* were compared to those for *hsGAL4* by *t*-test: *P* = 0.02, to *UAS-Imp-L2*: *P* = 0.05. (B) The levels of IMP-L2 protein were determined in whole flies by western blot, with actin as the loading control. The averages and standard errors of three independent measurements of IMP-L2 protein normalised to actin are given below the images, with the average of pooled controls set to one. The levels in *hsGAL4 > UAS-Imp-L2* were significantly different from the pooled controls (*P* = 0.04) or *UAS-Imp-L2* (*P* = 0.01) but not from *hsGAL4*, by *t*-test. (C) Levels of IMP-L2 protein were determined in haemolymph by western blot. All flies carried the *hsGAL4* driver with or without *UAS-Imp-L2* and were either kept at 25°C or placed at 37°C for 2 h prior to collection of haemolymph. Full genotypes of the flies tested were: *w*^*−*^/*w*^*−*^; *hsGAL4*/+; +/+, *w*^*−*^/*w*^*−*^; +/+; *UAS-Imp-L2*/+, *w*^*−*^/*w*^*−*^; *hsGAL4*/+; *UAS-Imp-L2*/+. Note that in A and B, the differences between the two controls were not statistically significant.

We first determined if increasing the amount of *Imp-L2* can modulate IIS in adult flies at a molecular level. *Imp-L2* is expressed in multiple tissues during development and in the adult (Osterbur *et al.*, 1988; Garbe *et al.*, 1993; Honegger *et al.*, 2008), and so we chose to initially over-express it ubiquitously. Since a strong ubiquitous over-expression of *Imp-L2* is lethal (Honegger *et al.*, 2008), we used the genetically weaker *UAS-Imp-L2* transgene created by Honegger *et al.* (2008) and drove its expression with a *heatshockGAL4* (*hsGAL4*) driver at 25°C. This manipulation resulted in viable flies and an 80% increase in the levels of *Imp-L2* mRNA in the adult female (Fig. 1A), as detected by qPCR. To monitor protein levels, we raised an antibody against recombinant IMP-L2 protein and affinity purified the serum. On western blots, we confirmed the previously observed apparent molecular weight of IMP-L2 as ∼30 kDa (Garbe *et al.*, 1993), and detected ∼80% increase in IMP-L2 protein in *hsGAL4 > UAS-Imp-L2* flies compared to the pooled average of the two controls (Fig. 1B), an increase equivalent to the mRNA increase. The details of mRNA and protein quantification methods are given in Experimental procedures.

IMP-L2 is predicted to be a secreted protein (Honegger *et al.*, 2008). Indeed, tagged IMP-L2 was efficiently secreted from S2 cells (data not shown). To confirm this *in vivo*, we looked for the native IMP-L2 in circulation. We could detect the protein in the adult haemolymph, both using antibodies previously described (data not shown) (Garbe *et al.*, 1993) and the antibody we generated (Fig. 1C). *hsGAL4* driven over-expression did not result in substantial increases in circulating IMP-L2 (Fig. 1C), probably because of low levels of induction at 25°C. To check that IMP-L2 protein could, in principle, be secreted from the transgene that we used, we increased the level of induction of *hsGAL4* by incubating the flies at 37°C for 2 h. This process resulted in a ∼8-fold increase in the mRNA over the *hsGAL4* control (data not shown) and a marked increase in the haemolymph IMP-L2 (Fig. 1C), indicating that IMP-L2 protein from the transgene could be correctly processed in our flies.

**Fig. 2:**
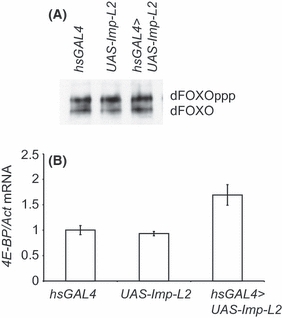
Increase in *Imp-L2* induces *4E-BP* transcript. (A) The phosphorylation of dFOXO in flies over-expressing *Imp-L2*. The phosphorylation was monitored as retardation on SDS-PAGE and dFOXO bands revealed by western blotting with an anti-dFOXO antibody. In separate wild-type extracts, the slower migrating band (dFOXOppp) was shown to disappear on treatment with calf intestinal phosphatase (data not shown). (B) The transcript levels of *4E-BP* were determined by qPCR, normalised to *Act* mRNA and the ratio in *hsGAL4* set to one. Means and standard errors are shown with *n* = 6 for *hsGAL4 > UAS-Imp-L2* and *n* = 7 for the two controls, *P* < 10^−3^ to either control by *t*-test, while the differences between the two controls were not significant. Full genotypes of the flies tested were: *w*^*−*^/*w*^*−*^; *hsGAL4*/+; +/+, *w*^*−*^/*w*^*−*^; +/+; *UAS-Imp-L2*/+, *w*^*−*^/*w*^*−*^; *hsGAL4*/+; *UAS-Imp-L2*/+.

To determine if this weak, ubiquitous over-expression of *Imp-L2* had an effect on molecular readouts of IIS status in the adult, we looked at the activation of the transcription factor thought to mediate the effects of IIS – dFOXO (Partridge & Bruning, 2008). The overall phosphorylation status of dFOXO, observed as slower migration on SDS-PAGE, reflects the activity of IIS in cell culture (Puig *et al.*, 2003). *In vivo* in the adult female, strong down-regulation of IIS also reduces levels of phosphorylated dFOXO in whole-fly extracts (Ikeya *et al.*, 2009). Over-expression of *Imp-L2* did not result in an observable difference in dFOXO phosphorylation (Fig. 2A), indicating that a strong down-regulation of IIS did not occur in our flies. Indeed, ablation of the median neurosecretory cells (mNSC) that produce DILP2, DILP3 and DILP5, a model of IIS reduction that increases adult lifespan (Broughton *et al.*, 2005), is also not enough to detectably alter whole-fly dFOXO phosphorylation status (data not shown). To detect more subtle changes in IIS, we examined the mRNA levels of *4E-BP*, a target of dFOXO (Junger *et al.*, 2003), which appears to mediate lifespan response to dietary restriction (Zid *et al.*, 2009), since low but prolonged activation of the transcription factor could result in an observable accumulation of the target message. Over-expression of *Imp-L2* led to a significant increase (∼80%) in *4E-BP* mRNA (Fig. 2B), consistent with activation of dFOXO. The data indicated that a subtle down-regulation of IIS occurs upon weak over-expression of *Imp-L2* in the adult.

### Secreted IMP-L2 binds native DILP2 and DILP5

IMP-L2 has been shown to interact with Flag-tagged DILP2 in insect cells (Arquier *et al.*, 2008; Honegger *et al.*, 2008). However, the increase in *4E-BP* mRNA observed on over-expression of *Imp-L2* is not observed upon deletion of only *dilp2*, but requires simultaneous deletion of *dilp2*, *dilp3* and *dilp5* (Gronke *et al.*, 2010). Therefore, it is likely that IMP-L2 can also bind DILPs other than DILP2, prompting us to seek evidence for a physical interaction. It was also important to establish whether IMP-L2 interacts with the native version of DILP2, because modified (Flag-tagged) DILP2 was used previously (Arquier *et al.*, 2008; Honegger *et al.*, 2008).

We wanted to examine the ability of IMP-L2 to bind native i.e. non-tagged and non-recombinant DILPs for two main reasons: the physical structure, including C-chain excision, of DILPs has not been characterised, precluding confirmation of the correct structure for a synthetic or recombinant DILP; and the presence of a tag may significantly alter the physical behaviour of DILPs because of their small size. To achieve this, we used a far-western blotting procedure, not requiring tagging or production of synthetic or recombinant proteins. To focus on DILP2, DILP3 and DILP5, we used proteins extracted from fly heads, where these DILPs are produced (Broughton *et al.*, 2005). We expressed myc epitope-tagged IMP-L2 in S2 cells and, to insure correct folding of the protein, we only used the secreted IMP-L2-myc_6_, in the form of conditioned medium, to probe female head proteins separated by non-reducing Tris–Tricine PAGE and transferred to a nitrocellulose membrane. IMP-L2-myc_6_ binding was then localised with an anti-myc antibody.

**Fig. 3:**
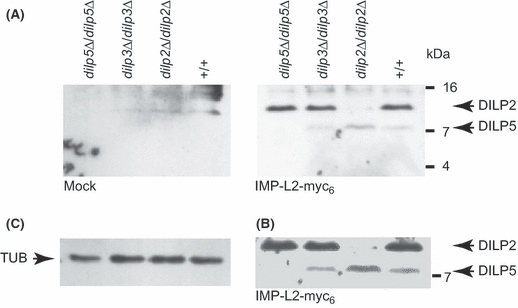
IMP-L2 binds DILP2 and DILP5. (A) Far-western blot was performed on head proteins from flies of the indicated genotypes using S2-cell conditioned media containing either IMP-L2-myc_6_ (right-hand panel) or not (mock; left-hand panel) and the binding of IMP-L2-myc_6_ visualised by an anti-myc antibody. Bands corresponding to DILP2 and DILP5 are indicated. (B) Higher exposure of the far-western shown above in A. (C) Tubulin used as loading control. Note that in all the panels the lanes shown align with the genotypes indicated in A. Full genotypes of the flies tested were: *w*^*−*^/*w*^*−*^; +/+; +/+, *w*^*−*^/*w*^*−*^; +/+; *dilp2Δ*/*dilp2Δ*, *w*^*−*^/*w*^*−*^; +/+; *dilp3Δ*/*dilp3Δ*, *w*^*−*^/*w*^*−*^; +/+; *dilp5Δ*/*dilp5Δ*.

The far-western blotting revealed IMP-L2 binding to two proteins of ∼8 and ∼12 kDa, and this specific binding was not observed in the mock control using conditioned medium from cells not expressing IMP-L2-myc_6_ (Fig. 3A,B). To identify the proteins bound by IMP-L2-myc_6_ we used head protein extracts from mutants deleted for each one of the three *dilp*s produced in the mNSC (Fig. 3A,B), and also probed the blots for DILP2 (not shown), and for tubulin as a loading control (Fig. 3C). The 12 kDa band co-migrated with the band recognised by the anti-DILP2 antibody (data not shown) and was absent in dilp2Δ/dilp2Δ flies (Fig. 3A,B), confirming this is indeed DILP2. Hence, secreted IMP-L2 can bind native DILP2. Note that a faint non-specific band, migrating close to DILP2 and present in dilp2Δ/dilp2Δ extracts, appears on the mock far-western blot (Fig. 3A) and is probably attributable to non-specific binding of the anti-myc antibody. The other specific band was absent in dilp5Δ/dilp5Δ (Fig. 3A,B), indicating that IMP-L2 can also interact with DILP5. The binding to DILP5 was weaker, and could be more easily observed on a higher exposure of the far-western blot (Fig. 3B), possibly because of lower levels of expression of *dilp5* compared to *dilp2* (Broughton *et al.*, 2005). Furthermore, there appeared to be more DILP5 in dilp2Δ/dilp2Δ fly heads (Fig. 3A,B), and this finding is consistent with the compensatory increase observed at the level of mRNA (Broughton *et al.*, 2008; Gronke *et al.*, 2010).

### Increased *Imp-L2* feeds back onto *dilp* expression

**Fig. 4:**
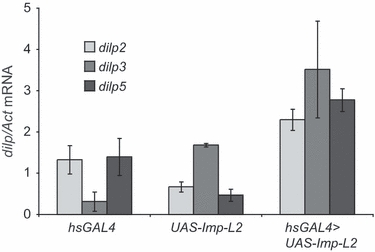
Increase in *Imp-L2* results in increased *dilp2*, *dilp3* and *dilp5* transcription. The levels of *dilp2*, *dilp3* and *dilp5* transcripts were determined by qPCR in whole fly RNA, normalised to *Act* mRNA and the average ratio of the two control genotypes set to one for each transcript. Means and standard errors are shown with *n* = 4 for all measurements except for *dilp5* in *UAS-Imp-L2* where *n* = 3. Two-way anova showed that the effect of genotype was significant: *P* < 10^−4^, where *hsGAL4 > UAS-Imp-L2* was different to both controls by *t*-test while the differences between the two controls were not significant, and there was no significant interaction between genotype and transcript. Full genotypes of the flies tested were: *w*^*−*^/*w*^*−*^; *hsGAL4*/+; +/+, *w*^*−*^/*w*^*−*^; +/+; *UAS-Imp-L2*/+, *w*^*−*^/*w*^*−*^; *hsGAL4*/+; *UAS-Imp-L2*/+.

As mentioned above, down-regulation or deletion of *dilp2* results in up-regulation of *dilp3* and *dilp5* transcription (Broughton *et al.*, 2008; Gronke *et al.*, 2010). Such feedback regulates in part the transcription of *dilp2*, *dilp3* and *dilp5*, and also encompasses *Imp-L2* (Broughton *et al.*, 2008; Gronke *et al.*, 2010): simultaneous deletion of *dilp2*, *dilp3* and *dilp5* results in a drop in *Imp-L2* transcript levels (Gronke *et al.*, 2010), suggesting a compensatory down-regulation of *Imp-L2* in response to IIS reduction. We wanted to examine if the inverse can also occur i.e. if increased levels of *Imp-L2* led to an elevation of *dilp* expression levels. Indeed, driving *Imp-L2* over-expression with the *hsGAL4* resulted in significant increases (∼2-fold) in the mRNA for *dilp2*, *dilp3* and *dilp5* (Fig. 4). Hence, *dilp2*, *dilp3* and *dilp5*, on one hand, and *Imp-L2* on the other, reciprocally regulate each other’s expression, forming a circuit in which changes in one are compensated by changes in the other.

### Up-regulation of *Imp-L2* increases lifespan

**Fig. 5:**
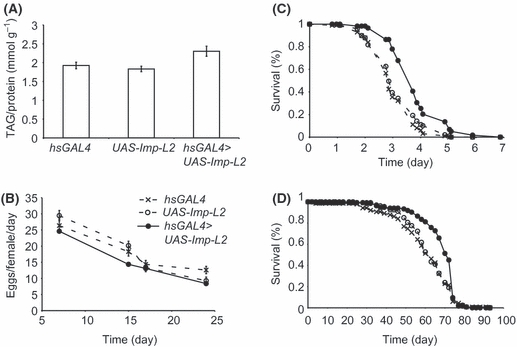
Over-expression of *Imp-L2* increases lifespan, oxidative stress resistance and whole-fly lipid content, while reducing fecundity. (A) Means and standard errors of the measurements of whole-fly triacylglycerol (TAG) per protein are shown, with *n* = 8 for each genotype, where *hsGAL4 > UAS-Imp-L2* is different to both controls by *t*-test (*P* = 0.01 to *hsGAL4*, *P* = 0.003 to *UAS-Imp-L2*). (B) The average number of eggs laid per female over 24 h was measured in ten separate vials per genotype at the times indicated. Means and standard errors are shown. Two Way anova showed that the effect of genotype was significant: *P* = 0.008, where *hsGAL4 > UAS-Imp-L2* is different to both controls by *t*-test; effect of time: *P* < 10^−4^; no interaction of the two main effects. Estimate of the cumulative eggs laid per female: 72 for *hsGAL4*, 72 for *UAS-Imp-L2* and 60 for *hsGAL4 > UAS-Imp-L2*. (C) Five-day old female flies (*hsGAL4 > UAS-Imp-L2 n* = 59, *hsGAL4 n* = 99, *UAS-Imp-L2 n* = 99) were placed on food containing 5% H_2_O_2_/suchrose and their survival determined over time. The survival of *hsGAL4 > UAS-Imp-L2* was significantly different from either control by Log-rank test (*P* < 10^−4^). (D) Lifespans of *hsGAL4 > UAS-Imp-L2* (*n* = 137, med = 71 days, max = 74 days) and the two genetic controls *hsGAL4* and *UAS-Imp-L2* (*n* = 142, med = 61 days, max = 74 days and *n* = 139, med = 61 days, max = 74 days). The survival of *hsGAL4 > UAS-Imp-L2* was significantly different from either control by Log-rank test, *P* < 10^−4^. Note in B, C and D the same symbols are used for the genotypes and are indicated in B. Full genotypes of the flies tested were: *w*^*−*^/*w*^*−*^; *hsGAL4*/+; +/+, *w*^*−*^/*w*^*−*^; +/+; *UAS-Imp-L2*/+, *w*^*−*^/*w*^*−*^; *hsGAL4*/+; *UAS-Imp-L2*/+. Note that in all panels the differences between the two controls were not statistically significant.

We next determined if weak, ubiquitous over-expression of *Imp-L2* was sufficient to cause an observable phenotype. IIS phenotypes tend to be more pronounced in female flies (Clancy *et al.*, 2001; Giannakou *et al.*, 2004; Hwangbo *et al.*, 2004; Broughton *et al.*, 2005), so we used female flies in our tests. IIS regulates metabolism in the adult, and down-regulation of IIS increases levels of stored lipids, whole body trehalose content and circulating sugars (Broughton *et al.*, 2005, 2008). Over-expression of *Imp-L2* caused a significant increase (21%) in the levels of stored lipids, as measured by determining whole-fly triacylglycerol content (Fig. 5A). On the other hand, while there was a trend towards an increase in the whole-fly trehalose content and the levels of circulating trehalose, glucose or the combined sugars, these were not significantly altered (Fig. S1A,B). Furthermore, an increase in starvation resistance was observed in only one of two trials performed (Fig. S1C). Therefore, apart from an increase in stored lipids, the effect of increasing *Imp-L2* on metabolic traits was not robust.

On the other hand, increasing *Imp-L2* did clearly affects other IIS-regulated traits. Over-expression of *Imp-L2* resulted in slight, but significant, reduction (17%) in cumulative eggs laid by an average female fly per day over the first 25 days of adult life (Fig. 5B), showing that *Imp-L2* could reduce fecundity. To examine their resistance to oxidative stress, we fed the flies 5% H_2_O_2_/sucrose food. The flies over-expressing *Imp-L2* survived for significantly longer (Fig. 5C), with a 23% increase in median survival time, indicating that increasing *Imp-L2* increases oxidative stress resistance. Most importantly, over-expression of *Imp-L2* using the *hsGAL4* driver significantly extended the lifespan of female flies at 25°C (Fig. 5D), with median lifespan extended by 15%, while the maximum lifespan remained unchanged with this driver. Note that, similar to over-expression of *dfoxo* (Giannakou *et al.*, 2004), *hsGAL4 > UAS-Imp-L2* had no effect on male lifespan (Fig. S2).

### Adult-onset over-expression of *Imp-L2* can extend lifespan

**Fig. 6:**
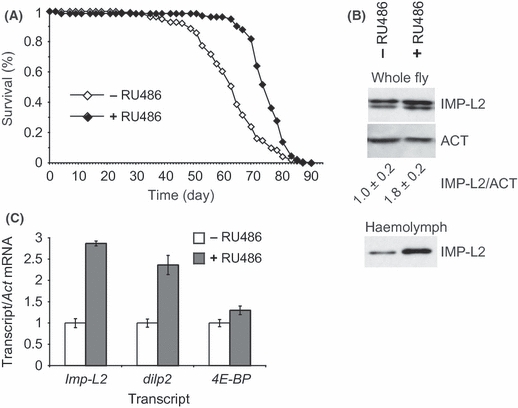
Adult-onset induction of *Imp-L2* increases lifespan. (A) Lifespans of female *ActGS > UAS-Imp-L2* flies induced to ubiquitously over-express *Imp-L2* by feeding RU486-containing food from day 3 of adulthood (+RU486, *n* = 143, med = 75 days, max = 84) or the uninduced controls (−RU486, *n* = 143, med = 63 days, max = 79 days). The survival of *ActGS > UAS-Imp-L2*+ RU486 was significantly different from the −RU486 control by Log-rank test (*P* < 10^−4^). Maximal lifespan was also extended upon *Imp-L2* over-expression (*P* < 0.05 by Log-rank test on the final 10% survivors). (B) Levels of *Imp-L2*, *4E-BP* and *dilp2* mRNA relative to *Act* mRNA were determined by qPCR in *ActGS > UAS-Imp-L2* female flies after 4 days of induction or in the uninduced control. Means and standard errors are shown, with values for the uninduced control set to 1 and with *n* = 7 for all measurements except for *Imp-L2* mRNA in +RU486 where *n* = 6. In each case −RU486 was significantly different to +RU486 by *t*-test (*Imp-L2*: *P* < 10^−4^, *dilp2*: *P* = 5 × 10^−4^, *4E-BP*: *P* = 0.04). (C) The levels of IMP-L2 protein were determined in whole flies (top) or in haemolymph (bottom) by western blot. For whole-fly extracts, actin was used as the loading control, and the averages and standard errors of three independent measurements of IMP-L2 protein normalised to actin are given below the images, with the levels in the uninduced control set to one; these were different by *t*-test (*P* = 0.04). Full genotype of *ActGS > UAS-Imp-L2* flies: *w*^*−*^/*w*^*−*^; *ActGS*/+; *UAS-Imp-L2*/+.

The *hsGAL4* driver is expressed in both the pre-adult and adult periods, and the lifespan-extension upon over-expression of *Imp-L2* achieved with this driver could thus be attributed to a developmental effect. To examine adult-onset over-expression, we used the inducible *Actin GeneSwitch* (*ActGS*) driver. *ActGS* drives ubiquitous transgene expression but only in the presence of the RU486 steroid drug (Ford *et al.*, 2007). Addition of RU486 to food had no effect on lifespan of *ActGS* or *UAS-Imp-L2* controls (Fig. S3), while in *ActGS > UAS-Imp-L2* adult female flies it almost doubled the period where no deaths were observed and resulted in a 20% increase in median lifespan, as well as a smaller increase in maximal lifespan (Fig. 6A), demonstrating that effect of *Imp-L2* on lifespan can be separated from its developmental effects.

Interestingly, while *ActGS > UAS-Imp-L2* female flies showed an almost 3-fold increase in *Imp-L2* mRNA upon RU486 feeding (Fig. 6B), the levels of IMP-L2 protein were only 80% increased (Fig. 6C). This apparent block to translation is indicative of translational control that appears exerted on both the native *Imp-L2* and the transgene we used (Honegger *et al.*, 2008). The reason why the *ActGS* driver had a more substantial effect on lifespan than *hsGAL4* may be because, in the case of the latter, the levels of IMP-L2 were increased in circulation (Fig. 6C). Adult-specific ubiquitous induction of *Imp-L2* also resulted in significantly increased levels of *dilp2* and *4E-BP* mRNA (Fig. 6B), as well as a decrease in fecundity (Fig. S4A) and an increase in H_2_O_2_ resistance (Fig. S4B). However, the increase in *4E-BP* observed here (∼30%, Fig. 6B) was lower than with *hsGAL4* (∼80%, Fig. 2B), indicating that prolonged IIS down-regulation or down-regulation during development may be required for a pronounced effect on *4E-BP* expression, and that the magnitude of lifespan extension is not proportional to the levels of *4E-BP* mRNA.

### Tissue-specific over-expression of *Imp-L2* can extend lifespan

**Fig. 7:**
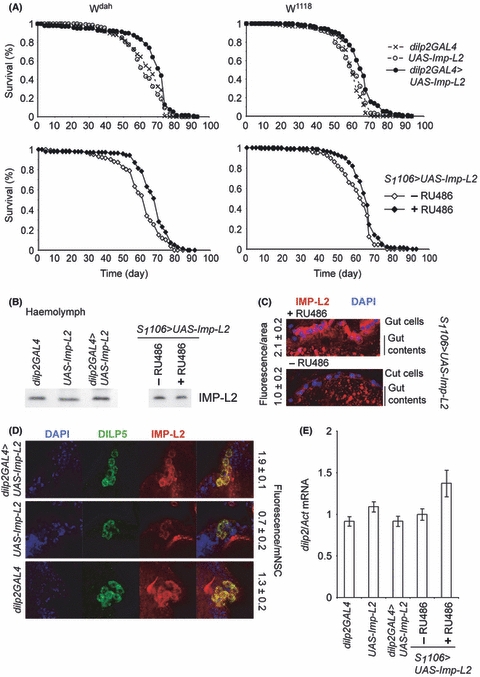
Tissue-restricted over-expression of *Imp-L2* increases lifespan. (A) Lifespans of female flies over-expressing *Imp-L2* in the mNSC (*dilp2GAL4 > UAS-Imp-L2*, *W*^dah^*n* = 142, med = 71 days, max = 77 days; *w*^1118^*n* = 141, med = 65 days, max = 72 days) and the two genetic controls (*dilp2GAL4*, *W*^dah^*n* = 149, med = 66 days, max = 74 days; *w*^1118^*n* = 140, med = 59 days, max = 67 days; *UAS-Imp-L2*, *W*^dah^*n* = 142, med = 66 days, max = 74 days; *w*^1118^*n* = 142, med = 59 days, max = 67 days; top two panels) or female flies in which over-expression of *Imp-L2* in the gut and fat body by the *S*_*1*_*106* driver was induced at day 3 of adulthood by RU486 (*S*_*1*_*106 > UAS-Imp-L2*+ RU486, *W*^dah^*n* = 143. med = 69 days, max = 77 days; *w*^1118^*n* = 144, med = 65 days, max = 74 days) or the uninduced controls (*S*_*1*_*106 > UAS-Imp-L2*−RU486, *W*^dah^*n* = 143, med = 62, max = 74; *w*^1118^*n* = 150, med = 63 days, max = 70 days; lower two panels), in outbred *W*^dah^ (two panels on the left) and the inbred *w*^1118^ (two panels on the right). The survival of *dilp2GAL4 > UAS-Imp-L2* was significantly different from the two controls in both backgrounds by Log-rank test (*P* < 10^−4^). Log-rank test also showed the survival of *S*_*1*_*106 > UAS-Imp-L2* significantly altered by addition of RU486, with *P* < 10^−6^ for *W*^dah^, *P* = 0.003 for *w*^1118^. In all cases, the maximal lifespan was extended upon *Imp-L2* over-expression (*P* < 0.05 by Log-rank test on the final 10% survivors). (B) IMP-L2 was measured in the haemolymph of *dilp2GAL4 > UAS-Imp-L2* female flies and the two genetic controls, or in *S*_*1*_*106 > UAS-Imp-L2* female flies fed or not with RU486, by western blotting. (C) IMP-L2 was visualised with immunofluorescence in the guts of *S*_*1*_*106 > UAS-Imp-L2* female flies fed or not with RU486. IMP-L2 is indicated in red, DAPI-stained nuclei in blue. Note the red fluorescence of the gut contents. The numbers next to the images give the mean and standard error of relative fluorescence intensity per unit area of gut as quantified, after background subtraction, from at least three animals (*P* = 0.01 by *t*-test). (D) IMP-L2 was visualised with immunofluorescence in the mNSC of wandering third-instar *dilp2GAL4 > UAS-Imp-L2* larvae or the two genetic controls. mNSC were identified with an anti-DILP5 antibody (green), IMP-L2 is indicated in red, DAPI in blue. The numbers next to the images give the mean and standard error of relative fluorescence intensity per mNSC quantified, after background subtraction, and averaged over at least three cells from four animals (*n* = 4, *t*-test *dilp2GAL4 > UAS-Imp-L2* to *dilp2GAL4 P* = 0.04, to *UAS-Imp-L2 P* = 0.001). (E) Levels of *dilp2* mRNA relative to *Act* mRNA were determined in the flies of the indicated genotypes/treatments, with *n* = 6 and the levels set to 1 in relevant controls. The levels in *S*_*1*_*106 > UAS-Imp-L2* were significantly altered by addition of RU486 (*t*-test: *P* = 0.05). Full genotypes of the flies used: *w*^*−*^/*w*^*−*^; +/+; *dilp2GAL4*/+, *w*^*−*^/*w*^*−*^; +/+; *UAS-Imp-L2*/+, *w*^*−*^/*w*^*−*^; +/+; *dilp2GAL4*/*UAS-Imp-L2*, *w*^*−*^/*w*^*−*^; *S*_*1*_*106*/+; *UAS-Imp-L2*/k. Note that in all cases the differences between *dilp2GAL4* and *UAS-Imp-L2* controls were not statistically significant.

Since IMP-L2 is a secreted protein, we were interested in determining if tissue-restricted over-expression can extend lifespan. In both the larvae and the adult, IMP-L2 is produced in distinct cells of both brain hemispheres (Honegger *et al.*, 2008). However, driving *UAS-Imp-L2* expression with the pan-neuronal *elavGAL4* driver (Luo *et al.*, 1994) did not extend lifespan (Fig. S5). IMP-L2 is also produced in the *corpora cardiaca*, part of the ring gland (Honegger *et al.*, 2008), but driving *UAS-Imp-L2* with the *corpora cardiaca*-specific *akhGAL4* driver (Kim & Rulifson, 2004) also failed to extend lifespan (Fig. S5). IMP-L2 is also expressed in the mNSCs, together with DILP2, DILP3 and DILP5 (Honegger *et al.*, 2008). Driving *UAS-Imp-L2* expression with the *dilp2GAL4* driver, expressed only in the mNSCs starting from the third-instar larval stage (Broughton *et al.*, 2005), significantly extended lifespan of female flies (Fig. 7A), prolonging the median survival time by ∼10%. The magnitude of the extension was similar to the one observed with *hsGAL4*, suggesting that the majority of the effect on lifespan of ubiquitous over-expression can be recapitulated by increased expression at the site of DILP2, DILP3 and DILP5 production.

IMP-L2 is also expressed in the fly gut and fat body (Honegger *et al.*, 2008). The *S*_*1*_*106* driver activates expression in these tissues upon addition of RU486 steroid drug to food (Poirier *et al.*, 2008). Adult-onset induction of *S*_*1*_*106 > UAS-Imp-L2* also significantly extended female fly lifespan (Fig. 7A), while addition of RU486 to food of *S*_*1*_*106* or *UAS-Imp-L2* controls had no effect (Fig. S3). Hence, IMP-L2 production in the gut/fat body can also contribute to longevity. With both the *dilp2GAL4* and *S*_*1*_*106* drivers, the maximum lifespan was also extended (Fig. 7A), confirming that Imp-L2 over-expression can extend maximum lifespan. In both cases, the enhanced longevity was observed in two different fly strains, the outbred *W*^dah^ and the inbred *w*^1118^ (Fig. 7A), indicating that it is robust to genetic background.

Similar to the situation in *hsGAL4 > UAS-Imp-L2*, the level of circulating IMP-L2 was not substantially increased in *S*_*1*_*106 > UAS-Imp-L2* flies fed RU486-containing food, or in *dilp2GAL4 > UAS-ImpL2* compared to controls (Fig. 7B). On the other hand, there were detectable increases in IMP-L2 protein in the gut of *S*_*1*_*106 > UAS-Imp-L2* flies in presence of RU486 (Fig. 7C; no significant increase was detectable in the fat body, Fig. S6), or in the mNSC of the third-instar *dilp2GAL4 > UAS-Imp-L2* larvae compared to controls (Fig. 7D), confirming the induction of the transgene. Note that certain other cells in the brain normally express much higher levels of IMP-L2 than those attained in the mNSC with *dilp2GAL4* (Fig. S7), so that the contribution from mNSC to the total pool of IMP-L2, even in the *dilp2GAL4 > UAS-Imp-L2* flies, is likely to be negligible, and not proportionate to its effect on lifespan. Hence, the site of expression, rather than the levels of IMP-L2, is relevant.

Tissue-specific induction of Imp-L2 appeared to selectively target lifespan and not other IIS regulated traits since, with both *dilp2GAL4* and *S*_*1*_*106* drivers, there was no effect on fecundity, stress resistance or *4E-BP* expression (Fig. S8). Intriguingly, the levels of *dilp2* mRNA were only increased when *Imp-L2* was induced in the gut/fat body, and not when it was induced in the mNSC (Fig. 7E), indicating that the feedback to *dilp* expression may occur via the former tissue(s).

## Discussion

In this study we determined the function of *Imp-L2* in adulthood, and found that its over-expression caused phenotypic changes consistent with negative regulation of IIS. Importantly, we found that this genetic manipulation of IIS could extend lifespan, which is not the case for all IIS-targeted interventions (Clancy *et al.*, 2001; Tatar *et al.*, 2001; Hwangbo *et al.*, 2004; Selman *et al.*, 2008; Ikeya *et al.*, 2009). It will be interesting to determine if this longevity-enhancing role of *Imp-L2* can be performed by an IGFBP or an IGFBP-rP, such as IGFBP7, in mammals. IGFBP7 has been characterised as a tumour suppressor, acting as a secreted senescence/apoptosis factor (Wajapeyee *et al.*, 2008). Establishing and examining the role of IGFBP7 in lifespan may shed light on the relationship between cellular senescence, cancer and whole organism aging in mammals, an important emerging field of study (Campisi & Yaswen, 2009).

Our data are consistent with IMP-L2 regulating IIS by sequestering the DILP ligands. In this respect, it is important that our binding assay was performed with native DILP proteins, revealing for the first time that IMP-L2 can bind native DILP5 as well as native DILP2. Interestingly, we did not observe binding to any other fly head proteins, despite the expression of DILP3 and DILP4 in the head (Ikeya *et al.*, 2002; Broughton *et al.*, 2005; Gronke *et al.*, 2010), implying that IMP-L2 can discriminate amongst different DILPs. However, this lack of observable binding may have also resulted from differential levels of expression of native DILPs e.g. from the very low levels of expression of *dilp3* in the mNSC (Broughton *et al.*, 2005). IMP-L2 can probably also bind DILPs other than DILP2 and DILP5, because strong over-expression of the protein is lethal (Honegger *et al.*, 2008), but simultaneous deletion of *dilp2*, *dilp3* and *dilp5* is not (Gronke *et al.*, 2010). The *dilp2Δ/dilp2Δ dilp3Δ/dilp3Δ dilp5Δ/dilp5Δ dilp6Δ/dilp6Δ* quadruple mutant is lethal (Gronke *et al.*, 2010), indicating that IMP-L2 may bind DILP6. Alternatively, IMP-L2 may also have DILP-independent effects, in a similar way that some IGFBPs appear to have IGF-independent functions (Mohan & Baylink, 2002).

Interestingly, sequence analysis indicates that DILPs are cleaved and processed like insulin (Gronke *et al.*, 2010), however, no study to date has physically observed these processed forms of DILPs. While we have previously observed DILP2 on a western blot (Broughton *et al.*, 2008), this is the first time that the native DILP5 has been observed on an SDS-PAGE. For both of these DILPs, the apparent molecular weight is too large for the processed DILPs (predicted molecular weight of DILP2 and DILP5 processed like insulin is ∼6 kDa) and is closer to the predicted molecular weight of the pro-peptide or an uncleaved IGF-like peptide (∼13 and ∼10 kDa for DILP2 and DILP5 respectively). IMP-L2′s ability to bind these uncleaved forms is consistent with its binding to human pro-insulin, IGF-I and IGF-II, as well as insulin (Sloth Andersen *et al.*, 2000), also indicating that these uncleaved forms of DILPs may be functional in the fly.

Removal of *dilp2*, *dilp3* and *dilp5* results in down-regulation of *Imp-L2* transcription (Gronke *et al.*, 2010), and we show here that, reciprocally, up-regulation of *Imp-L2* results in up-regulation of the mRNAs for all three *dilp*s. This compensatory feedback loop is, in both cases, not sufficient to completely correct the disturbance of the fly IIS status since both genetic manipulations result in phenotypes consistent with down-regulation of IIS including lifespan extension. Interestingly we find that over-expression of *Imp-L2* in the mNSC does not result in increased *dilp2* mRNA, indicating that this feedback does not occur via an/a autocrine/paracrine mechanism. Rather, the feedback occurs via effector(s) produced in the gut/fat body, since up-regulation of *Imp-L2* in these tissues resulted in increased *dilp2* transcript levels. Hence, peripheral tissues, such as the fat body, are not only in charge of regulating DILP release from the mNSC in response to nutritional changes, as has been observed in larvae (Geminard *et al.*, 2009), but are also main regulators of *dilp* synthesis in response to changes to adult IIS status.

Our study indicates that an agent present in circulation has an effect on lifespan; a finding that may have important therapeutic applications. Indeed, the genetic manipulation that led to increased IMP-L2 levels in the haemolymph (*ActGS > UAS-Imp-L2*+ RU486) was the one that resulted in the most substantial increase to longevity. Interestingly, in other cases where we could observe an extension of lifespan, we did not observe a substantial increase in the levels of circulating IMP-L2. It is possible that IMP-L2 was secreted but then retained in a target tissue, so that no net increase was observed in circulation. Alternatively, IMP-L2 may have predominantly acted in an/a autocrine/paracrine manner. In either case, IMP-L2 could have been retained in specific tissue(s) based on its interactions with components of extracellular matrix or cell surface proteins, as is known for IGFPBs (Hwa *et al.*, 1999; Mohan & Baylink, 2002). In the case of *dilp2GAL4 > UAS-Imp-L2*, the over-expression of IMP-L2 might have been very efficient in sequestering DILPs at the site of their production. In the case of *S*_*1*_*106* driven over-expression, the gut may be the most relevant tissue since it is here that we could observe a substantial increase in IMP-L2. Increased activity of dFOXO using the same *S*_*1*_*106* driver is sufficient to extend lifespan (Giannakou *et al.*, 2004), and the gut may be the relevant tissue in this case as well. Interestingly, out of all the IIS-regulated adult traits, lifespan was most sensitive to increased *Imp-L2*, since no other phenotypes examined were responsive to tissue-specific *Imp-L2* induction.

## Experimental procedures

### Fly stocks and husbandry, phenotypic tests

*UAS-Imp-L2* this is the genetically weaker transgene generated by Honegger *et al.* (2008), *heatshockGAL4* (Bloomington Stock Center), *dilp2GAL4* (Broughton *et al.*, 2005), *S*_*1*_*106* (Giannakou *et al.*, 2004), *ActGS* (Ford *et al.*, 2007), *dilp2Δ/dilp2Δ*, *dilp3Δ/dilp3Δ* and *dilp5Δ/dilp5Δ* (Gronke *et al.*, 2010) were backcrossed at least six times into the outbred Dahomey background carrying the *w*^1118^ mutation (*W*^dah^) (Giannakou *et al.*, 2004), which had been cured of the *Wolbachia* infection; or the inbred *w*^1118^ background, which is *Wolbachia* free. Note that the *ActGS* line used (255B) is thought to have multiple insertions of the driver (Ford *et al.*, 2007), however, after more than six backcrosses we did not observe any segregation of different dye colours. All experiments were performed at 25°C, 12-hour light/dark cycle, and controlled humidity, on female flies in *W*^dah^ background, unless otherwise noted. Flies were reared at standard density on SYA food (5% sucrose, 10% yeast, 1.5% agar), female flies sorted on day 3 and kept ten per vial. Where required, induction by RU486 was performed as described previously (Giannakou *et al.*, 2004). For starvation, flies were kept on 1% agar, and for H_2_O_2_ treatment on food containing 1% agar, 5% sucrose, 5% H_2_O_2_, starting on day 5. For RNA or protein extraction the flies were frozen in liquid nitrogen on day 7. Lifespan experiments, whole-fly trehalose, circulating sugar (Parrou & Francois, 1997; Broughton *et al.*, 2008) or whole-fly lipid (Gronke *et al.*, 2005) quantifications were performed as described.

### Antibody production, western blots and immunofluorescence

cDNA coding for IMP-L2 without the signal peptide was amplified from *W*^dah^ cDNA using the primers: CACCAGAGCCGTGGACCTGGTAGACG and TTAGTCTTCCTCATTAAGTACGGGA-TAC, cloned into pENTR/D-TOPO vector (Invitrogen, Paisley, UK), sequenced and transferred into pDEST17 vector (Invitrogen) adding a His_6_ tag. The protein was expressed in BL21(DES3) *E. coli*, recovered in the insoluble fraction and purified under denaturing conditions on Ni-NTA agarose (Quagen, Crawley, UK), followed by preparative SDS-PAGE. Anti-IMP-L2 antibody was raised in rabbits by Eurogentec (Eurogentec, Fawley, UK), and affinity purified against rIMP-L2. Fly protein extractions and western blots were performed as described (Giannakou *et al.*, 2007; Broughton *et al.*, 2008). Affinity purified anti-Imp-L2 was used at 1:2000 dilution. For quantification, the blots were developed with ECL, images captured with LAS-1000 cooled CCD (Fujifilm; Fujifilm UK Ltd, Bedfordshire, UK) and band intensities determined with ImageJ (freeware from Research Services Branch, National Institute of Mental Health, Bethesda, Maryland, USA). The quantity of IMP-L2 was expressed relative to ACT, with this ratio set to one in the control genotypes/untreated flies. Haemolymph was extracted from 7-day old female flies as described (Broughton *et al.*, 2008) and 1 μL used for western blots. Images presented were taken on film. For immunofluorescence, flies were dissected in ice-cold PBS and stained as described (Broughton *et al.*, 2010) with the affinity purified anti-IMP-L2 antibody at 1:1000 dillution and TexasRed conjugated secondary antibody, and co-stained where required with rat anti-DILP5 and AlexaFluor488 conjugated secondary antibody as described (Broughton *et al.*, 2010). Images were captured on Zeiss LSM 700 (Carl Zeiss Ltd, Hertfordshire, UK), and quantified using ImageJ.

### Far-western blotting

The last two exons of Imp-L2, which include the signal peptide, were amplified from *W*^dah^ genomic DNA with the primers: CACCATGAATTTACATGTGTGCGCCTTAG and GTCTTCCTCATT-AAGTACGGGATAC, cloned in to pENTR/D-TOPO vector, sequenced and transferred into pTWM (giving *UAS*_*t*_*-Imp-L2-myc*_*6*_). S2 cells were cultured in serum-free medium (Invitrogen) and co-transfected using Cellfectin (Invitrogen) with plasmids encoding either *Act5C-GAL4*, *UAS*_*p*_*-GFP* and *UAS*_*t*_*-Imp-L2-myc*_*6*_ (producing IMP-L2-myc_6_) or *Act5C-GAL4* and *UAS*_*p*_*-GFP* (mock control), and the conditioned media harvested after 3 days. Hundred micrograms of fly-head proteins were separated on non-reducing Tris–Tricine PAGE and transferred to nitrocellulose membranes as described (Broughton *et al.*, 2008). The membranes were blocked in PBST (PBS + 0.2% Tween-20) with 5% BSA, probed over-night with a 1 in 10 dilution of the conditioned media in the same buffer at 4°C and IMP-L2-myc_6_ binding visualised with an anti-myc (Sigma, Dorset, UK) western blot.

### qPCR

RNA extraction, cDNA synthesis and qPCR were performed as described (Broughton *et al.*, 2008), except for *dilp* qPCR which was performed on 7900HT Fast Real-Time PCR System using Fast Syber Green Master Mix (Applied Biosystems). The *Act*, *dilp2*, *dilp3* and *dilp5* primers have been described (Broughton *et al.*, 2008). The following primers were used for *Imp-L2*: CCTCATTAAGTACGGGATAC and CTTCTGATCTCCGAGATCAAG; for *4E-BP*: CACTCCTGGAGGCACCA and GAGTTCCCCTCAGCAAGCAA. The amount of the relevant mRNA was expressed relative to *Act* mRNA and this ratio set to one in the control genotypes/untreated flies.

### Statistical analysis

Statistical analysis was performed either in jmp (SAS, Cary, NC, USA) or Excel. Details are given in figure legends.

## Supporting Information

**Fig. S1** Whole body trehalose content, circulating sugars and starvation resistance in *hsGAL4 > UAS-Imp-L2* flies.

**Fig. S2** Life spans of *hsGAL4 > UAS-Imp-L2* male flies.

**Fig. S3** Lack of effect of RU486 addition to *ActGS, UAS-Imp-L2* or *S1106* controls.

**Fig. S4** Reduced fecundity and increased H_2_O_2_ resistance upon induction of *Imp-L2* with the *ActGS* driver.

**Fig. S5** Lifespans of *elavGAL4 > UAS-Imp-L2* and *akhGAL4 > UAS-Imp-L2* flies.

**Fig. S6** IMP-L2 produced in the fat body of induced *S_1_106 > UAS-Imp-L2* adult female flies.

**Fig. S7** IMP-L2 distribution in larval brains.

**Fig. S8** Lack of effect on fecundity, H_2_O_2_ resistance and *4E-BP* expression upon induction of *Imp-L2* with the *dilp2GAL4* or the *S_1_106* driver.

As a service to our authors and readers, this journal provides supporting information supplied by the authors. Such materials are peer-reviewed and may be re-organized for online delivery, but are not copy-edited or typeset. Technical support issues arising from supporting information (other than missing files) should be addressed to the authors.
