# Supplementary material for: Lifespan extension by increased expression of the Drosophila homologue of the IGFBP7 tumour suppressor
Source: Aging Cell. 2011 Feb;10(1):137–47. doi: 10.1111/j.1474-9726.2010.00653.x (PMC3042147; doi:10.1111/j.1474-9726.2010.00653.x)

SUPPLEMENTAL FIGURE CAPTIONS

Supplemental Figure 1 Whole body trehalose content, circulating sugars and starvation resistance in *hsGAL4>UAS-Imp-L2* flies. A Means and standard errors of the measurements of whole-fly trehalose with n=10. No significant difference was detected by t-test. **B** Levels of haemolymph trahalose and glucose were determined and are expressed here as glucose equivalent total sugar. Means and standard errors are shown, with n=10 and no significant differences by t-test. **C** Starvation assay trials on *hsGAL4>UAS-Imp-L2* flies and their controls. The assays were started with 100 flies. *hsGAL4>UAS-Imp-L2* were significantly different from both controls only in the 2nd trial, by Log-rank test ($p<10^{-3}$).

Supplemental Figure 2 Lifespans of *hsGAL4>UAS-Imp-L2* males. Lifespans were started with 150 male flies. *hsGAL4>UAS-Imp-L2* males were not longer lived than either of the two controls.

Supplemental Figure 3 Lack of effect of RU486 addition to *ActGS*, *UAS-Imp-L2* or *$S_1$106* controls. Lifespans were started with 150 female flies. There were no significant differenced detected by Log-rank test.

Supplemental Figure 4 Reduced fecundity and increased H_2O_2 resistance upon induction of *Imp-L2* with the *ActGS* driver. A The average number of eggs laid per female over 24 h was measured in 10 separate vials in presence or absence of RU486 at the times indicated. Means and standard errors are shown. Two Way ANOVA showed that the effect of RU486 was significant (p=0.0003), as well as the effect of time ($p<10^{-4}$) and the interaction of the two main effects (p=0.03). **B** After 4-day treatemnt with RU486 100 seven-day old females were placed on 5% H_2O_2/suchrose and their survival determined over time. Effect of RU was significant (Log-rank test p=0.03).

Supplemental Figure 5 Lifespans of *elavGAL4>UAS-Imp-L2* and *akhGAL4>UAS-Imp-L2* flies. Lifespans were started with 150 female flies.

Two *elavGAL4* insertion lines were used, one on the X chromosome the other on the 2nd. There were no significant differences detected by Log-rank test.

Supplemental Figure 6 IMP-L2 produced in the fat body of induced *$S_1$106>UAS-Imp-L2* adult females. IMP-L2 was visualised with immunofluorescence using anti-IMP-L2 antibody in the fat body of *$S_1$106>UAS-Imp-L2* adult flies fed or not with RU486.

Supplemental Figure 7 IMP-L2 distribution in larval brains. IMP-L2 (red) was visualised with immunofluorescence using anti-IMP-L2 antibody in the brains of 3rd-instar wondering larvae of the indicated genotypes. Brains were co-stained with anti-DILP5 (green) and DAPI (blue). mNSCs are indicated with a grey arrow. Cells strongly expressing IMP-L2 and whose projections pass close by the mNSCs are indicated with a white arrow.

Supplemental Figure 8 Lack of effect on fecundity, H_2O_2 resistance and *4E-BP* expression upon induction of *Imp-L2* with the *dilp2GAL4* or the *$S_1$106* driver. **A** and **B** The average number of eggs laid per female over 24 h was measured in 10 separate vials for **A** the *dilp2GAL4>UAS-Imp-L2* flies and their controls or **B** the *$S_1$106>UAS-Imp-L2* adult flies fed or not with RU486, at the times indicated. Means and standard errors are shown. Two Way ANOVA showed no significant effect of genotype in **A**, or RU486 in **B**. **C** 100 seven-day old females were placed on 5% H_2O_2/suchrose and their survival determined over time. *$S_1$106>UAS-Imp-L2* females were fed RU486 or not for 4 days prior to the stress. No significant differences were detected by Log-rank test. **D** The levels of *4E-BP* mRNA relative to *Act* mRNA were determined in the whole-fly RNA with qPCR and the levels in the pooled controls set to one. No significant difference was detected by t-test.

Supplemental Figure 1

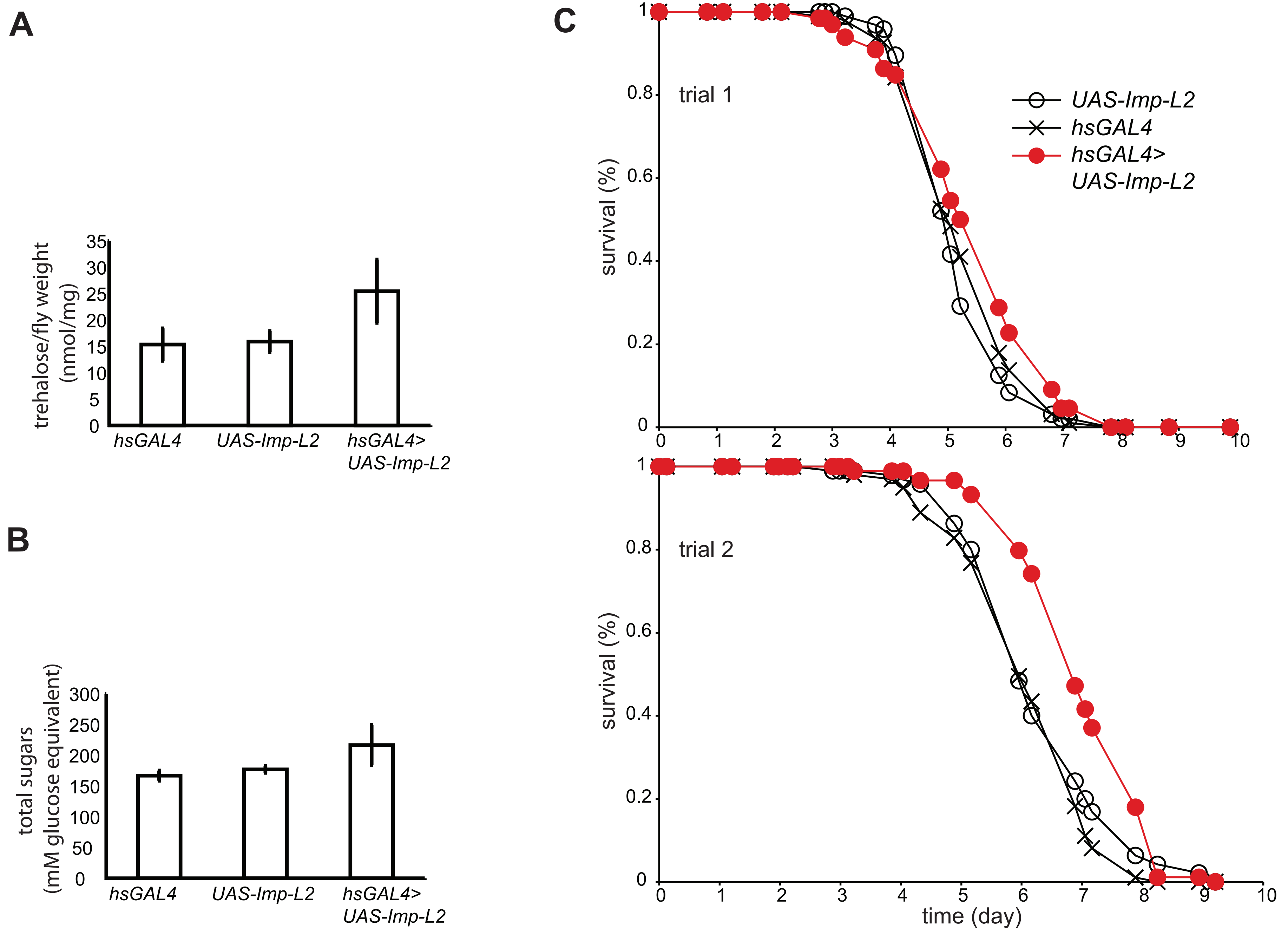

Supplemental Figure 2

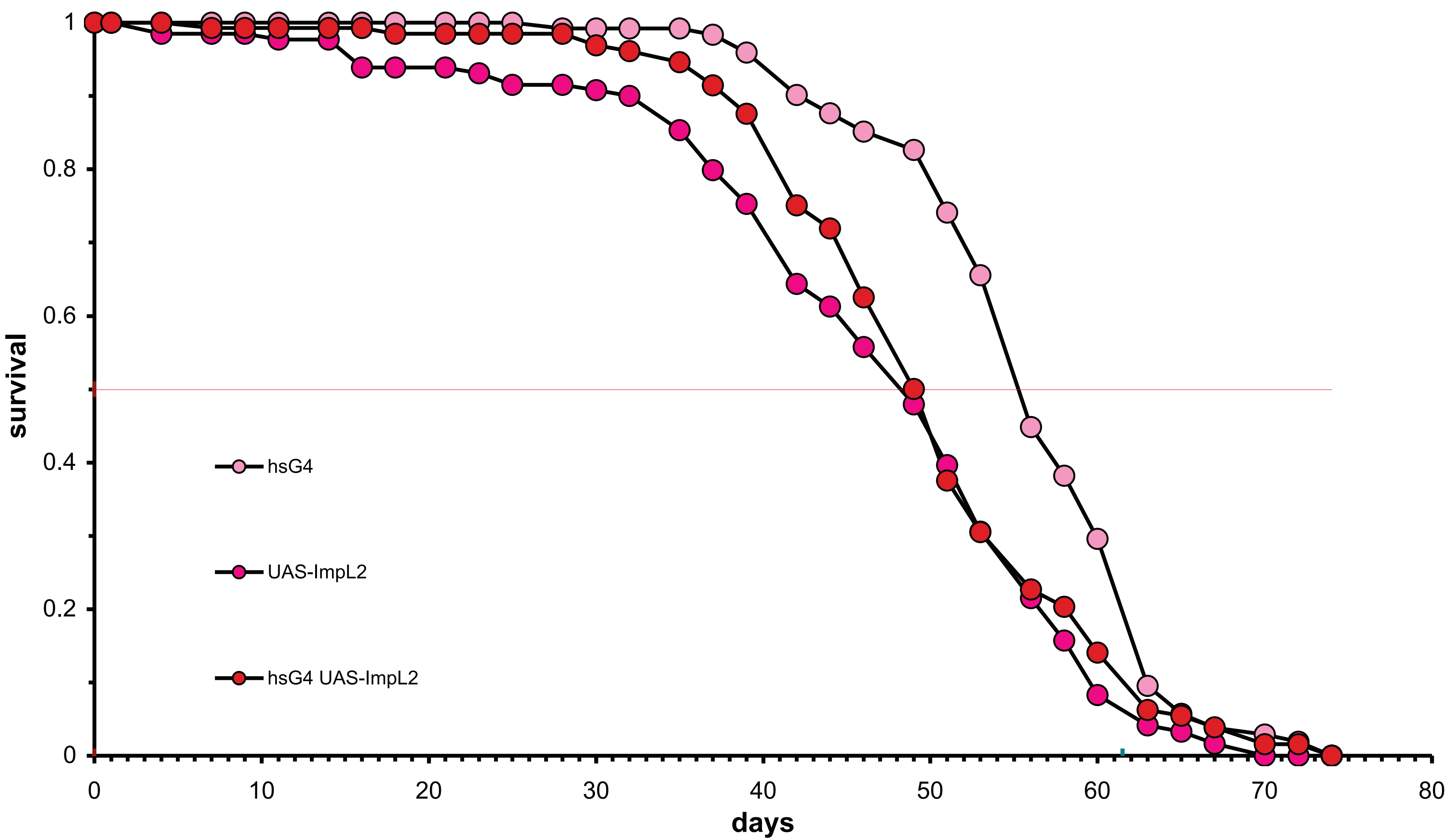

Supplemental Figure 3

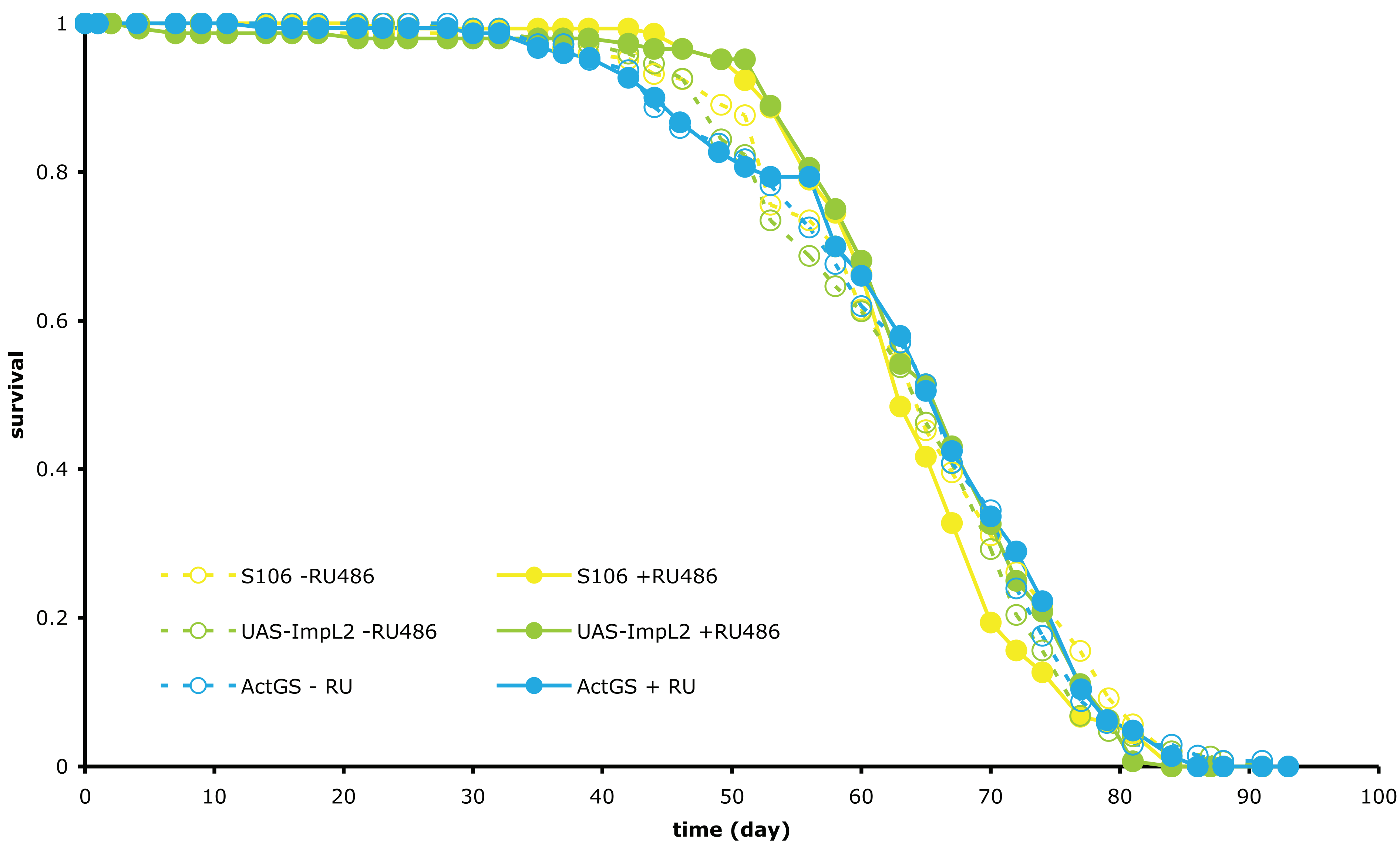

Supplemental Figure 4

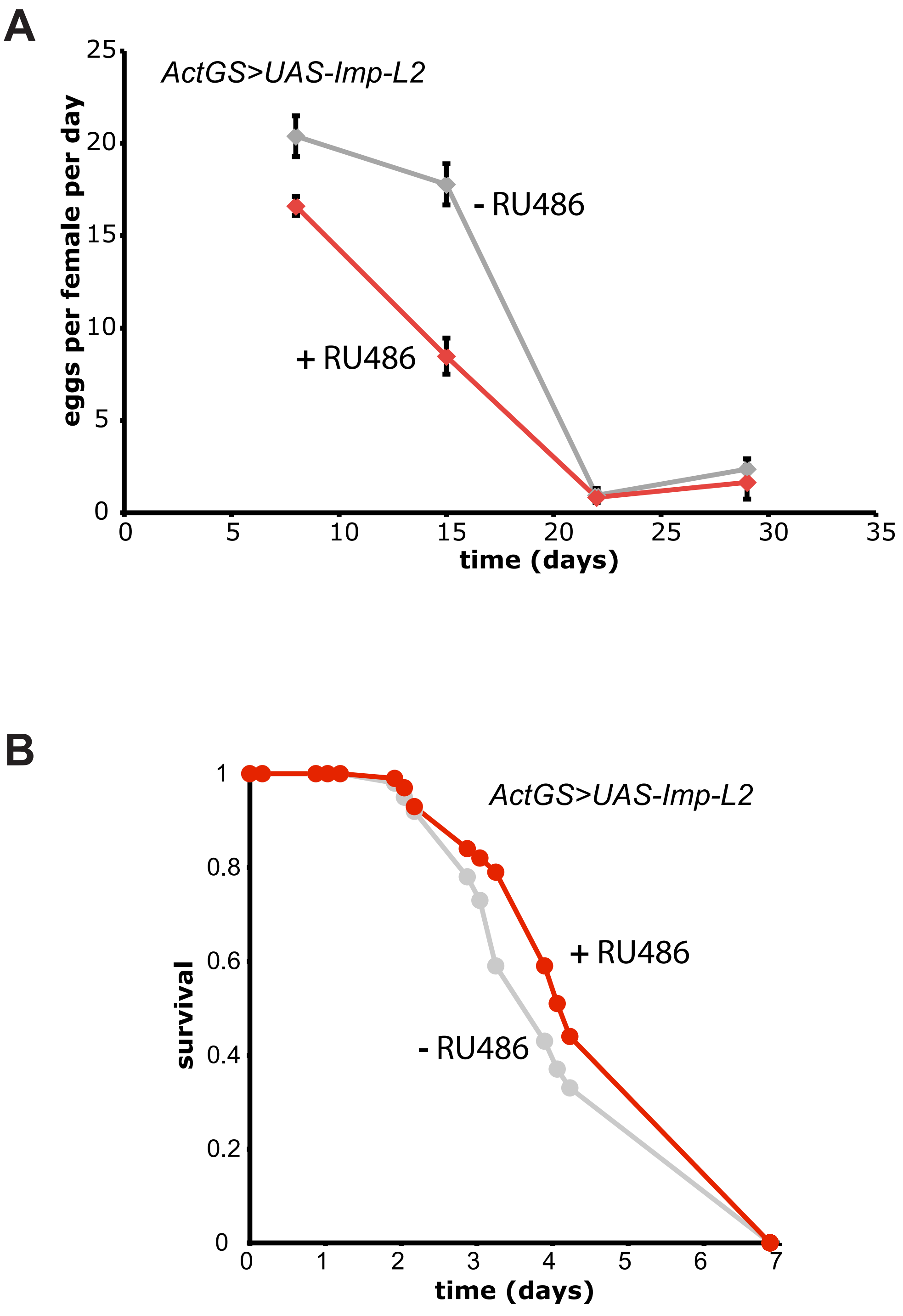

Supplemental Figure 5

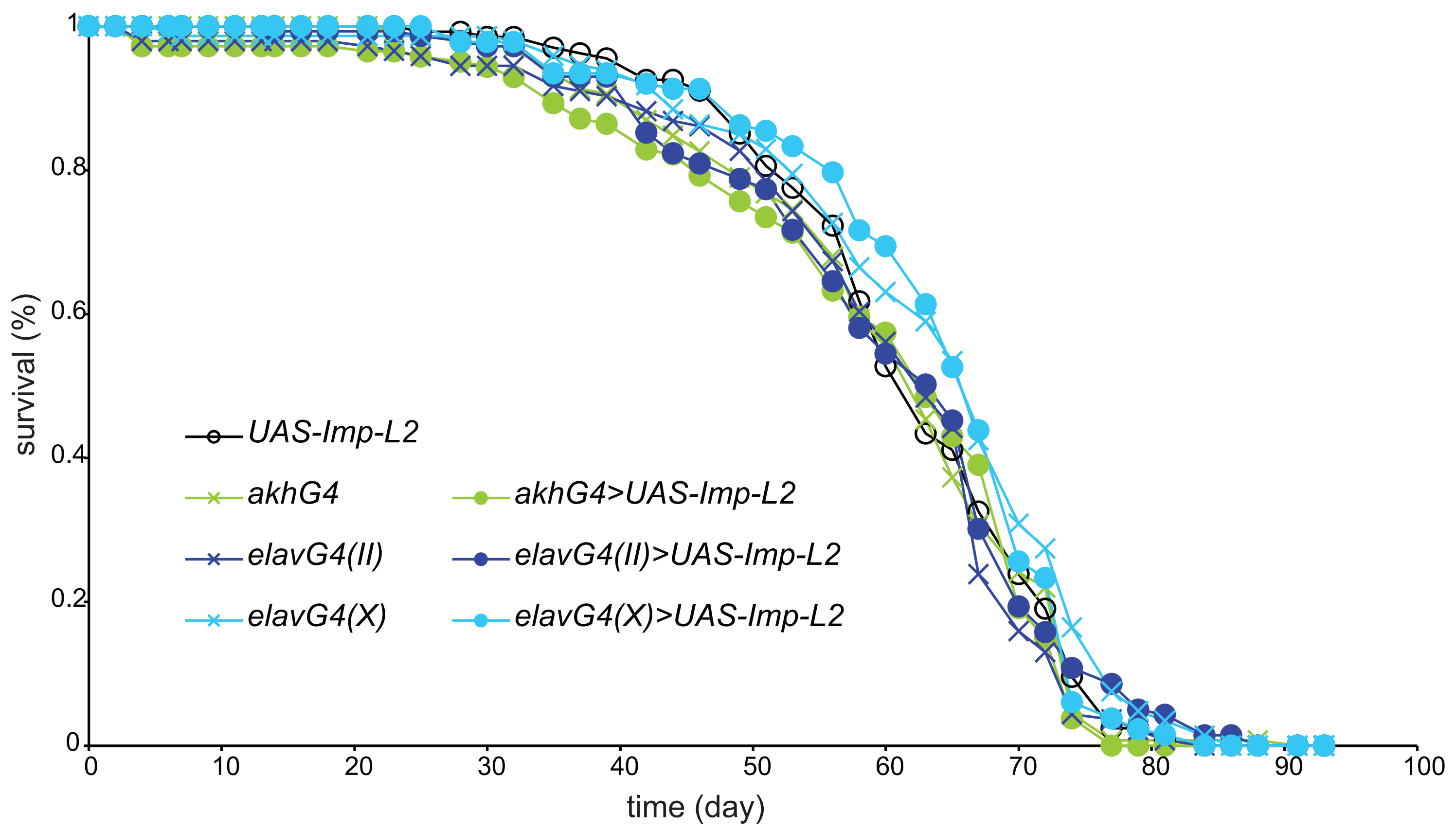

Supplemental Figure 6

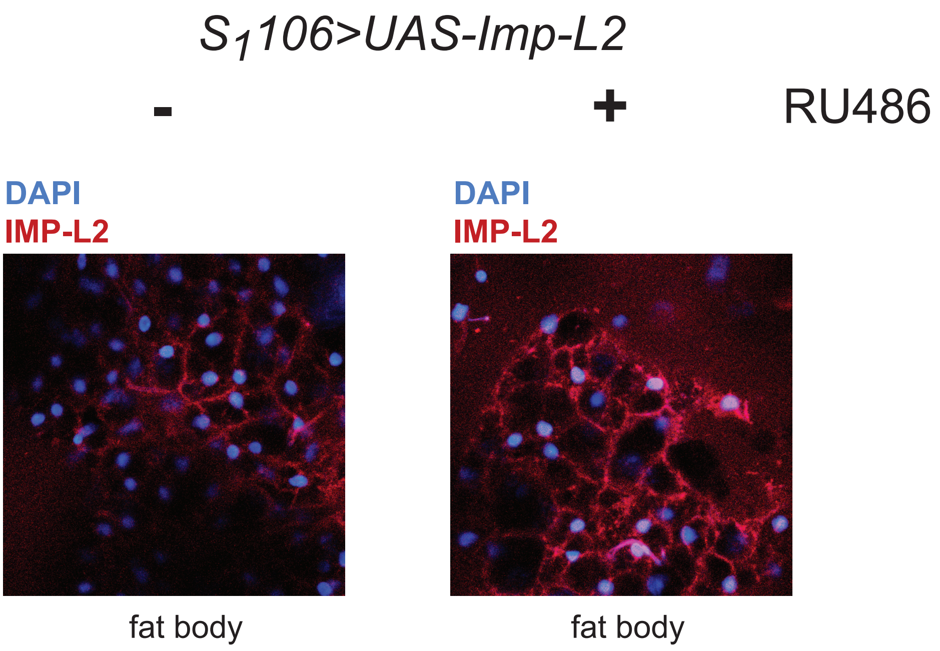

Supplemental Figure 7

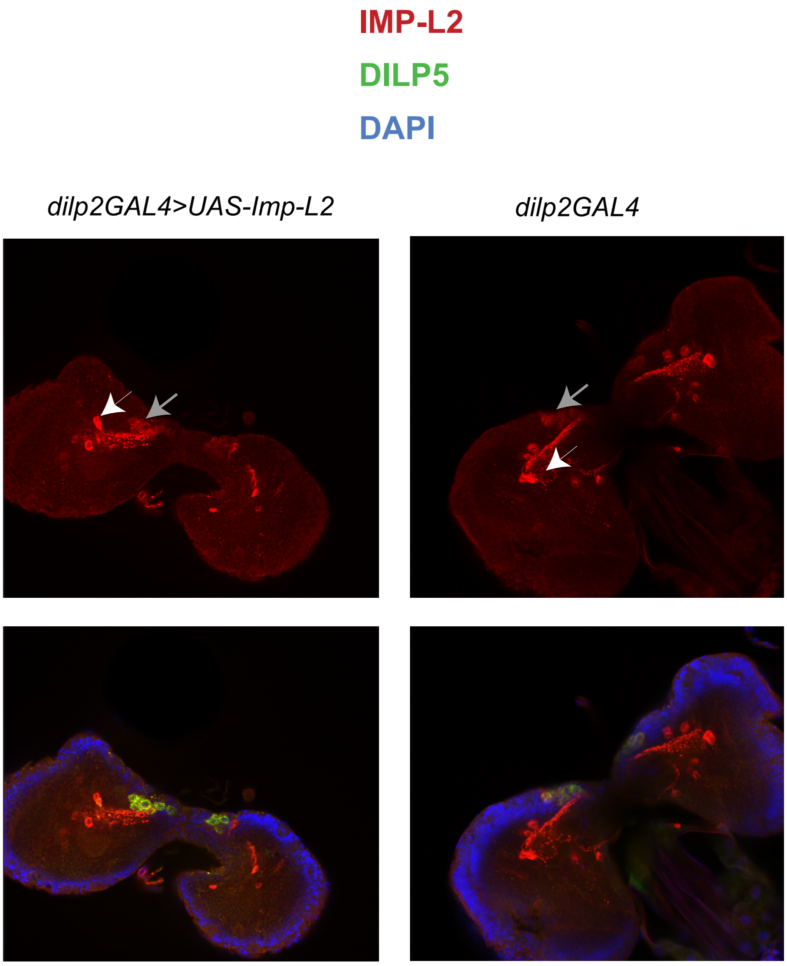

Supplemental Figure 8

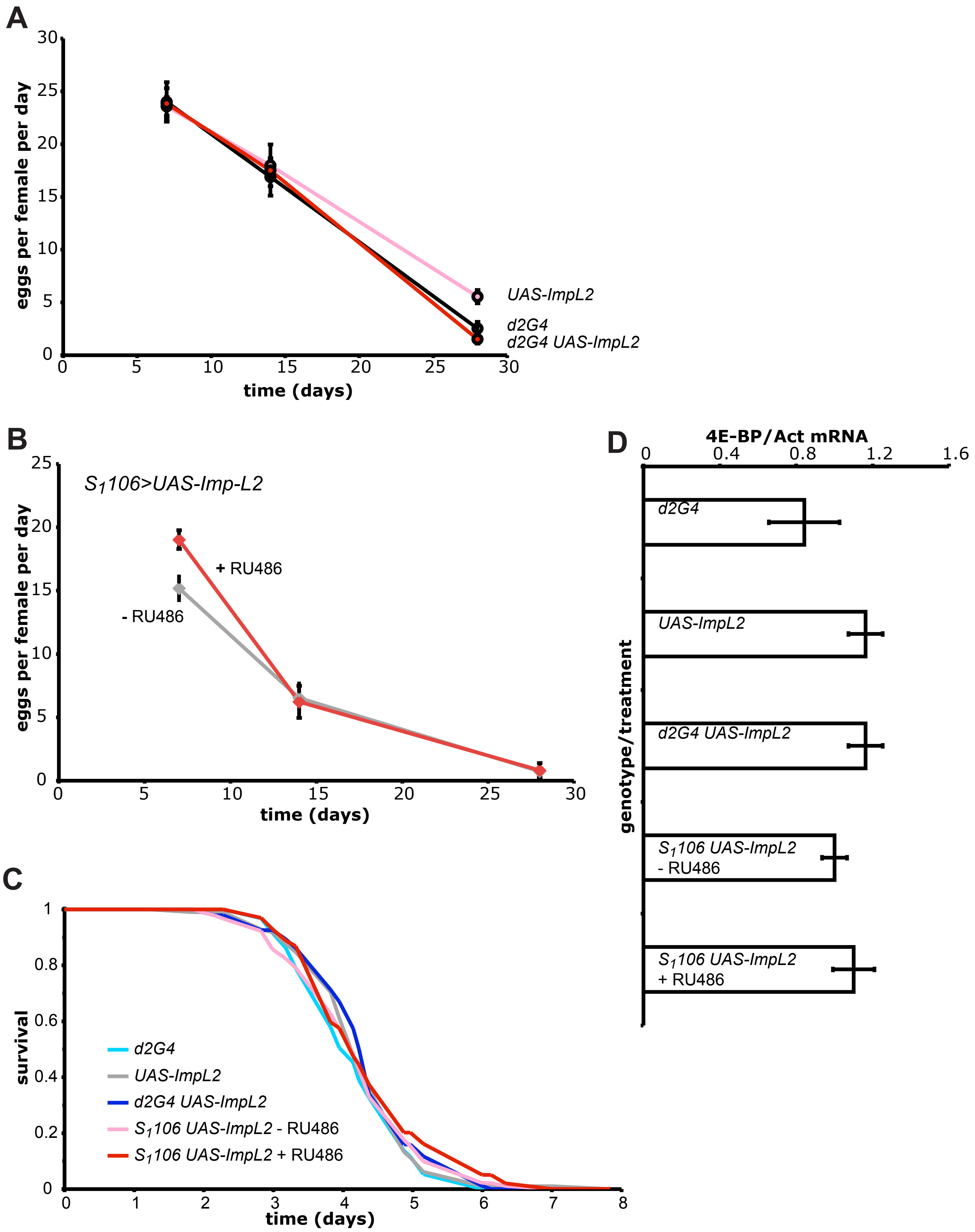

Supplement: Supplementary file 4 [file acel0010-0137-SD4.pdf]
